# 

**Published:** 1892-02

**Authors:** 


### Notice.

The Post-Graduate Dental Association, of the United States, will hold its annual meeting April 29th and 30th next, at Leland Hall, Chicago.

Dr. N. C. Barrett, of Buffalo, N. Y.; Drs. T. N. Brophy, Louis Ottafy and others, of Chicago, will present essays and addresses. An interesting programme has been arranged and a good attendance is expected. All members of the profession are invited.

Graduates of recognized Dental Colleges may become members by paying membership fee $1.00, and dues for one year in advance $1.00.

L. S. Tenney, Sec'y.
R. B. Tuller, Pres. 96 State Ave., Chicago.

### Hymeneal.

Dr. Edward P. Beadles and Miss Annie Boisseau were married recently in Danville, Virginia, at the residence of the bride's father.

Dr. Beadles is one of the more prominent dentists of Virginia, and occupies an enviable position not only in his profession but in society as well. Dr. and Mrs. Beadles will have the congratulations and the best wishes of his host of friends in the profession, and we are sure that the new relationship thus formed will be a happy one, and that Dr. Beadles will more than ever stand as one of the representative men of his chosen profession.

### EDITORIAL.

### The Mississippi Valley Dental Association.

The forty-eighth annual meeting of this society will be held in Cincinnati, March 8th to the 11th. The Executive Committee is making special effort for an unusually good meeting, and, without doubt, their effort will be crowned with success.

This society, though encroached upon somewhat by other societies, is always an attractive meeting, especially for all the older members. It is interesting not only because of what it accomplishes from year to year, but because of its history. It was organized when dentistry could hardly be regarded as a profession, or at least, when it was in a very elementary condition; but two or three dental societies were in existence in the world at that time, and they have ceased to exist. This society has not only lived through this long period, but it has been one of the active agencies in the unfolding and development of many things that are now important elements in the constitution of our profession. With its early history many of the leading members of the profession were identified, and made their impress upon its work; the results of which remain to the present. Many prominent teachers, writers and workers have been members of this society and received much of their training within it.

The executive committee, who are distributed throughout the various neighboring States, are at work in the preparation of a programme, and the coming meeting will doubtless be one of the most interesting that has been held for years. Here little or no time is spent in routine work but the time is almost wholly devoted by papers and discussions upon scientific and professional subjects.

We trust there will be a large attendance, and that all the members will come with full intent to add something to the interest and importance of the occasion.

### CONTENTS.

COMMUNICATIONS.

A MONTHLY MAGAZINE DEVOTED TO THE INTERESTS OF THE

### DENTAL PROFESSION.

*Terms Reduced to* $2.00 *Per Year. Single Copies,* 25 *Cents.*

### EDITED BY PROF. J. TAFT, D.D.S.

—AND—

### Published by SAM'L A. CROCKER & CO., Ohio Dental and Surgical Depot.

The volume commences in January and closes in December.

The REGISTER being issued monthly is always fresh, therefore Indispensable to the practitioner who desires to keep posted up with the new inventions and improvements which are daily developed. Neither expense nor effort will be spared to give value and variety to its contents. Articles will be illustrated with well executed engravings whenever they will add to the interest or assist to explanation.

Its extensive circulation and frequency of issue make it one of the BEST DENTAL ADVERTISING MEDIUMS in the United States.

All subscriptions must begin with January or July.

Local or special notices, five lines or less, will be inserted for $3 each insertion; over five lines, 50 cts. per line.

All advertising bills payable in advance, or at furthest, after the issue of the first number containing the advertisement. All transient advertisements to be paid for in advance.

☞CONTRIBUTIONS SOLICITED.

Exclusively Editorial Communications should be addressed to the Editor.

The latest number of the REGISTER may be ordered with or without other goods at any time, and all letters should be addressed to

SAM'L A. CROCKER & CO., Ohio Dental and Surgical Depot, Cincinnati, O.

